# ‘Taking the green pill’: An interpretative phenomenological analysis of the lived experiences of climate distress

**DOI:** 10.1111/papt.70052

**Published:** 2026-03-11

**Authors:** Jessica L. Morgan, James D. Gregory, Marc O. Williams

**Affiliations:** ^1^ School of Psychology, Cardiff University Cardiff UK

**Keywords:** climate distress, eco‐anxiety, eco‐distress, interpretative phenomenological analysis, moral identity

## Abstract

**Introduction:**

Climate distress (CD) is an emerging psychological response to the climate crisis, encompassing anxiety, grief, shame, and helplessness. While empirical research has begun to explore its prevalence and emotional impacts, little is known about the lived experience of CD. This study qualitatively explored how CD is experienced, maintained, and managed.

**Methods:**

Semi‐structured interviews were conducted with 10 participants experiencing CD, recruited via social media. Data were analysed using interpretative phenomenological analysis (IPA), allowing for an in‐depth exploration of participants' experiences.

**Results:**

Three superordinate themes were identified: (1) moral selves in crisis; (2) climate distress is all‐consuming; and (3) finding relief. CD was closely tied to participants' moral identity, often resembling features of moral injury and perfectionistic striving. Distress was intensified by perceived powerlessness, unmet ethical standards, and concern that relief from distress would signal moral disengagement. Value‐driven actions provided meaning and relief but were frequently accompanied by shame, burnout and emotional exhaustion. Psychological support was sometimes experienced as invalidating when moral and contextual dimensions of CD were not acknowledged.

**Conclusions:**

CD is a morally grounded response to an ongoing and existential global threat, shaped by identity, values and wider socio‐political contexts. Supportive responses should avoid individualising or pathologising distress, instead attending to how responsibility is understood and internalised, and to how individuals relate to their distress in ways that allow for sustainable moral engagement. Therapeutic approaches that support individuals to hold responsibility with self‐compassion and within realistic limits of individual agency, alongside collective forms of support, may be particularly valuable.

## INTRODUCTION

We are living in an era of unprecedented climate and ecological change, resulting in direct mental health implications such as post‐traumatic stress from extreme weather events and experiences of distress from loss, displacement, and conflict (Cruz et al., 2020; Lawrance et al., 2021). Importantly, even the awareness of climate change can cause distress, which has become an area of increasing interest in psychology (Whitmarsh et al., 2022).

*Climate Distress* (CD) encompasses a wide range of emotional experiences, including anxiety, depression, grief, anger, and shame stemming from awareness of climate/ecological change (Williams, 2023). A variety of related terms are used in the literature to describe aspects of this experience, including eco/climate‐anxiety and solastalgia (Pihkala, 2022). For example, eco‐anxiety has been defined as ‘heightened emotional, mental or somatic distress in response to dangerous changes in the climate system’ (Climate Psychology Alliance, 2020, p. 22), highlighting the cognitive, physiological, and affective aspects.

Importantly, CD has been found to cause impairment to daily functioning and to present frequent social challenges (Clayton & Karazsia, 2020). Recent work has therefore begun to distinguish between the presence of climate‐related distress and the extent to which it interferes with functioning, for example through the Climate Change Distress and Impairment Scale (CC‐DIS; Hepp et al., 2023). Higher levels of CD and related impairment have been associated with poorer well‐being and reduced quality of life (Gago et al., 2024).

Although awareness of CD is growing, the literature remains in its infancy, with most studies published since 2022 (Jarrett et al., 2024). Research to date has been dominated by quantitative approaches, exploring prevalence and risk factors for CD (Hickman et al., 2021; Prencipe et al., 2023; Whitmarsh et al., 2022), coping strategies (Ojala, 2012a, 2012b), links with pro‐environmental behaviour (Lutz et al., 2023; Roberts et al., 2025) and developing appropriate measures (Clayton & Karazsia, 2020; Hogg et al., 2021). However, a systematic review by Ramsay et al. (2025) concluded that existing scales lacked content validity, as items are largely based on pre‐existing psychological constructs (i.e., what is already known about anxiety from other models and theories) rather than derived from qualitative accounts of lived experience.

Theoretical frameworks for CD have also begun to emerge. Marks et al. (2024) offered a cognitive behavioural account of CD, conceptualising it as arising from climate‐related events that trigger negative thoughts (e.g., hopelessness), leading to intense emotions (e.g., anxiety), physiological responses (e.g., exhaustion), and maladaptive behaviours (e.g., avoidance). These elements interact in a reinforcing cycle that contributes to clinically significant distress. Others have conceptualised CD as a rational and adaptive response: ‘practical anxiety’ (Kurth & Pihkala, 2022), or a moral response to existential threat (Sims et al., 2020). While these accounts provide useful lenses, they remain largely speculative in the absence of empirical work capturing how individuals themselves understand and live with CD. The qualitative literature exploring CD has begun to provide richer insights (e.g., worry for future generations, apocalyptic worries), but these have tended to focus on specific populations such as climate professionals, activists, and psychotherapy clients (Jarrett et al., 2024; Soutar & Wand, 2022). This study aims to add to the qualitative literature in a way that addresses these limitations.

While CD is not considered pathological (Bhullar et al., 2022), it can substantially affect well‐being, quality of life, and mental health. Interventions to support those experiencing CD are under‐developed and under‐researched, and professionals report feeling unprepared to respond (Baudon & Jachens, 2021; Hickman et al., 2021; Williams et al., 2024). Furthermore, while clinical psychology concerns itself with reducing distress and impairment, in the case of CD it is unclear whether distress should always be regarded as maladaptive, given its associations with higher rates of the kinds of environmental actions that are vital for reducing greenhouse gas emissions (Nielsen et al., 2024; Roberts et al., 2025; Whitmarsh et al., 2022). Advancing understanding of CD is therefore essential not only for phenomenological understanding and the development of appropriate clinical responses but also for shaping behaviour change interventions and informing public health policy.

## AIMS

This study aims to explore the subjective experiences of CD to gain a deeper understanding of its presentation and how it impacts the lives of those who experience it. The research question guiding this study was: *What is the lived experience of individuals experiencing climate distress?* In particular, we focused on the cognitive, emotional, and behavioral features of CD, as well as on factors that may maintain it, including how others are perceived and experienced in this context. We also explored the impact of CD on daily life.

## MATERIALS AND METHODS

### Research design and epistemological position

This qualitative study employed an Interpretative Phenomenological Analysis (IPA) to explore the subjective perspectives of adults who experience CD, as it is particularly suited for exploring complex and nuanced phenomena, allowing for an in‐depth exploration of participants' experiences and their interpretations (Eatough & Smith, 2017; Smith et al., 2009). The interpretative phenomenological epistemology that we adopted assumed that individuals engage in active meaning‐making, and that meanings are shaped by social, relational, and cultural contexts (Smith et al., 2009). Our focus was therefore on how participants understood the experience of CD within the contexts that give their experiences meaning.

To capture the richness and complexity of these experiences, semi‐structured interviews were chosen as the primary method of data collection. By employing IPA and semi‐structured interviews, this study ensures a context‐sensitive and participant‐centred approach, offering rich insights into how individuals experience and make sense of CD.

### Inclusion criteria

This study used a purposive sampling method to recruit participants that met the following inclusion criteria:

#### Inclusion

- At least 18 years of age.
- Self‐reported experience of CD, defined as any negative affect associated with the awareness of climate change and associated functional impairment (Clayton & Karazsia, 2020).
- At least one of these experiences of CD must have occurred in adulthood (i.e., age ≥ 18).
- If residing outside the UK, must be able to speak and understand English to a degree that allows engagement in nuanced and in‐depth semi‐structured interviews.
- Able to speak and understand English to the level required to engage in interviews.

### Recruitment and interview process

Participants were recruited between June 2024 and February 2025 through social media advertisement, reaching out to relevant groups and organisations (e.g., Greenpeace, Climate Psychology Alliance, Extinction Rebellion) and snowball sampling. These environmentally‐engaged and activist groups were approached in the expectation that there would be a high degree of CD, and as they were accessible through community networks. Participants accessed a Qualtrics survey via a link provided on a social media advert. The form presented the information sheet and consent form before collecting data to assess study eligibility. Eligible participants proceeded to a second survey in Qualtrics, which gathered demographic information and sought permission to contact them for potential interviews. Participants also completed a measure of climate change distress and impairment (the CC‐DIS; Hepp et al., 2023) as a means of situating the sample. The CC‐DIS has a response scale of 1–5 (from 1: strongly disagree to 5: strongly agree), and means/standard deviations were computed for each subscale. The CC‐DIS was deemed useful for its separate items relating to distress (covering anger, sadness, and anxiety) and impairment (general, social, and work/school) and for its psychometric properties: the distress scale has demonstrated excellent internal consistency across studies (α = .92–.93) and the impairment scale has acceptable‐to‐high internal consistency (α = .81–.89). There is support that the measure's two factor structure is moderately consistent across samples (Hepp et al., 2023).

Semi‐structured interviews were utilised as the method of qualitative data collection. The interviews were completed online using Microsoft Teams and were guided by an interview protocol developed based on the research questions, existing literature, and guiding IPA theory. The interviews were recorded through the Microsoft Teams app and then transcribed verbatim before analysis. Interviews ranged from 55 to 180 min in length (median 117 min), depending on the depth of discussion and participant engagement.

### Participants

Sample size was guided by IPA's commitment to idiography and depth of analysis. IPA recommends small, homogeneous samples that allow for detailed, case‐by‐case examination before moving to cross‐case interpretation, and the commonly suggested range is 6–10 participants (Smith et al., 2009). Considering the relatively narrow focus of our research aim – focussing on emotional, cognitive and behavioural dimensions of CD within a largely Western sociocultural context, we judged that 10 participants allowed us to maintain idiographic depth while capturing sufficient variation in the lived experience of CD.

The mean age of the sample was 36.3 (SD = 13.57), ranging from 23 to 61 years of age and contained six women and four men. The sample was highly educated: everyone had at least an undergraduate degree and some had an additional postgraduate degree. The sample included two neurodivergent participants, and three participants with a diagnosis of a mental health condition unrelated to CD, with one participant awaiting a mental health assessment. The majority of the sample resided in the United Kingdom (although many of the participants were born outside of the UK), and the rest of the participants resided in countries outside the UK. All demographic and clinical information (in relation to mental health and neurodivergence) were self‐reported. A demographic summary can be found in Table 1.

**TABLE 1:** Demographic summary of the sample (*N* = 10).

Demographic			
Age	23–61 years old (mean = 36.3 years old)		
		*n*	%
Gender	Women	6	60
	Men	4	40
Country of Residence	United Kingdom	5	50
	Colombia	2	20
	The Netherlands	1	10
	Canada	1	10
	Mexico	1	10
Highest Level of Education Achieved	Undergraduate degree or equivalent	4	40
	Postgraduate degree or equivalent	6	60
Clinical diagnosis indicative of neurodivergence	Yes	2	20
	No	8	80
Clinical diagnosis of a mental health condition	Yes	3	30
	No	6	60
	Currently being assessed	1	10

Participants demonstrated high levels of distress according to the CC‐DIS (Hepp et al., 2023) (M = 4.56, SD = 0.24) and moderate‐to‐high levels of impairment (M = 3.40, SD = 0.28), with low variability in both scales. In comparison, Hepp et al. (2023) reported moderate levels of distress across four separate general population groups (majority from continental Europe) sampled in 2021/2022 (Ms = 3.14 to 3.84, SDs = 0.26 to 0.90) and low to moderate levels of impairment (Ms = 1.96–2.58, SDs = 0.33–0.74). The findings indicate that this study's participants experience greater distress and impairment than reported in in Hepp et al.'s (2023) studies, and the lower variability in scores indicates more homogeneity in the current sample. A full summary of CC‐DIS scores can be found in the Tables S1–S2.

### Data analysis

IPA follows six phases: becoming immersed in the data (reading and re‐reading transcripts), initial coding (making descriptive comments), developing emergent themes (synthesising initial codes into short phrases that capture essential elements of meaning), searching for connections across emergent themes to develop superordinate themes, repeating the process with the next transcript (whilst also bracketing ideas from previous transcripts), and finally identifying patterns across all transcripts (Smith et al., 2009). Coding of papers and organisation of themes took place entirely by hand. The first author conducted the analysis, with reflexive auditing and theme checking by the third author to enhance credibility.

### Positionality and reflexivity

As the first author (JM), my interest in CD is both professional and personal. Professionally, I am interested in the rise of discussions about climate change in my direct clinical work and the lack of guidance for working clinically with people who experience CD. Personally, my concerns about climate change also shape my engagement with this topic. It is important to note that I have not experienced CD, but I engage in pro‐environmental behaviours due to my concerns about climate change.

Before starting this research, my position on CD was that it was a normal reaction to a frightening and real phenomenon. I had the opinion that some distress about climate change was adaptive for people to engage in pro‐environmental behaviour, but too much distress could be maladaptive and impairing.

I am not diagnosis‐focused in my clinical practice, instead often focussing on how our experiences impact our wellbeing. However, my therapeutic training has been largely based in cognitive behavioural therapy (CBT) and systemic family therapy, and I regularly integrate Acceptance and Commitment Therapy (ACT) into my practice; I tend to hold these models in the front of my mind when exploring a person's distress.

Whilst my aim was not to mitigate bias (Galdas, 2017), I remained mindful of my own perspectives throughout this study by keeping a reflective diary. This was helpful in logging my reflections and questions throughout the entire process, so that I could remain curious, open‐minded, and avoid over‐reliance on my own preconceptions to attain the most ‘authentic’ interpretation in the double hermeneutic process. I also used supervision for these purposes.

### Ethical considerations

This study was reviewed and approved by Cardiff University's Research Ethics Committee (reference number: EC.24.04.16.6998R). Research Ethics Committee. Informed consent was obtained from all participants who took part, and their data were anonymised using pseudonyms. Participants had the right to withdraw at any stage before data anonymisation, without needing to provide a reason. Participants were debriefed and signposted to support services at the end of each interview.

## RESULTS

The analysis yielded three superordinate themes: (1) Moral Selves in Crisis, (2) Climate Distress is All‐Consuming, (3) Finding Relief from Distress, and nine subordinate themes (see Table 2). Themes were closely related and interdependent. Themes and subthemes are described below and substantiated by excerpts of interviews.

**TABLE 2:** Summary of superordinate and subordinate themes.

Superordinate theme	Subordinate themes
Moral selves in crisis	Moral Identity
	Moral Injury
	Moral Perfectionism
Climate distress is all‐consuming	It's in Everything I Do
	Constant Stress
	Too Much for Others
Finding relief from distress	Experiences of Interventions
	Self‐Implemented Strategies
	Connection, Belonging, and Community

### Moral selves in crisis

This theme describes how morality, identity, moral injury, and perfectionism were core components of participants' experiences and central to the maintenance of CD through developing a sense of inner conflict.

#### Moral identity

Participants described a set of longstanding political and ethical values that were fundamentally connected to their experiences of CD. Their views were largely homogeneous, with shared concerns about inequality. Although passionate about various causes, participants consistently identified climate change as their foremost priority, viewing it as the ultimate existential threat:

I talk a lot about women's rights, and you know, I'm also worried about poverty and…[the] state of schools…none of this matters if we can't have a planet where we can live on. Lauren.

Morality was framed as central to participants' self‐concept. They viewed themselves as ‘good’, ‘caring’ and ‘responsible’ individuals who were highly empathetic towards those most vulnerable to the effects of climate change: ‘*I care about the people of the planet*…*I want everyone to be OK*’ (*Ryan*).

CD and caring about the planet – particularly out of concern for other people – became integral to participants' moral identity and, as a result, meant that they viewed CD as something that was unchangeable:

I think I took the green pill…Once you've seen that you can't unsee it. Billy.

The ‘green pill’ (a reference to the red pill/blue pill metaphor in popular culture), signified an irreversible transformation that fundamentally reshaped how participants perceived themselves and the world around them.

#### Moral injury

Participants' distress was often rooted in perceived violations of their moral code, either through witnessing or contributing to insufficient action on climate change and the perceived powerlessness in being able to create change. This misalignment, whether on a personal or systemic level, was described as deeply distressing, unethical, and consistent with the concept of moral injury (Litz et al., 2009). This is exemplified by Ryan, who felt ‘*very sorry for those people who are going to lose their homes, their livelihoods, family members, because of a problem that we all know is coming*’. Powerlessness was experienced by some in the context of perceiving powerful actors (‘*governments and leaders*’ [Ryan]) as not doing enough; ‘*The problem is the government*’ (Beth).

A profound sense of injustice appeared to underpin this moral distress, provoking strong emotional reactions such as anger and sadness: ‘…*feelings of injustice of things that we've done to non‐humans [and] ourselves will always come back to me. And, you know, that's something I can't shake off*’ (Jacqui).

Participants frequently reported feeling powerless in the face of the climate crisis, particularly in their inability to influence broader systems or societal responses, further contributing to moral injury: ‘…*because I cannot really help to stop the problem. I don't have the power. I don't think anybody can, I don't think that even collectively we can*.’ (*Jeff*).

For many, the experience of powerlessness itself was distressing, as it signified an acceptance of climate change and potential loss of moral engagement – something seen as incompatible with their core identity. Participants articulated a perceived need to resist feelings of powerlessness and to maintain ongoing engagement:

I feel hopeless, but I'm still hanging on…That's why I can't feel any kind of resolution or feeling of acceptance…if I do, then it's almost like we've lost the game. Lauren.

Despite its psychological toll, participants interpreted this moral distress as meaningful. Experiencing CD and moral injury reaffirmed their ethical standards and their identity as morally engaged individuals. For many, distress functioned as a necessary motivator for ongoing climate action: ‘*It feels like a necessary evil*…*Because we need to survive as a human species. So, I think it's good that it's there [climate distress]. It means something good about me. It means that I care. It means that I've not completely lost hope*.’ (*Lauren*).

#### Moral perfectionism

Participants adopted coping strategies centred on personal responsibility and continuous ethical action, helping restore a sense of agency. Although these behaviours were often helpful, many participants exhibited perfectionistic tendencies and binary thinking in relation to their moral conduct. This led participants to set high or unachievable standards for themselves, then experiencing distress when they were not able to meet these standards.

Even minor deviations from participants' high standards and moral code evoked shame, self‐criticism, and an ‘instant sense of guilt’ that ‘…just sat with me for days and it's quite horrible.’ (Jacqui).

Living according to these moral standards within a system that did not share the same priorities created a constant tension. Participants reported frequently facing ethical dilemmas with no ideal solution, leading to ongoing distress and moral compromise:

Like, the number of times where I'm in a supermarket and I have to choose between the organic apples…in plastic film or…not organic apples but…not in plastic. It's like what is the right choice?… at some point I still have to live. I still have to eat the damn apple. Lauren.

### Climate distress is all‐consuming

The pursuit of moral perfection led participants to increasing levels of engagement with climate‐related information and action. Disengagement, although potentially less painful in one sense, was perceived as ignorance and moral failure. As a result, participants remained engaged, despite the emotional cost. As a result, CD – and the behaviours adopted to manage it – permeated participants' everyday lives, consumed their thinking, and strained their relationships.

#### It's in everything I do

Participants reported that CD permeated every aspect of their lives, from daily routines to major life decisions. Many described significant lifestyle changes, which reinforced the sense of CD's omnipresence: ‘*Obviously [my lifestyle] changed a lot in order to help*……*[it's in] everything I do*’ (*Jacqui*).

Some participants made life‐altering choices, such as abstaining from air travel, adopting a vegan lifestyle, or choosing not to have children, in an effort to tightly align with their moral commitments. These choices both came as a result of being consumed by CD and also contributed to its all‐consuming nature. Jamie described being ‘*fully committed*’ to the cause, having ‘*transformed my life*’ and ‘*sterilised myself*’: ‘*How can I do anything else when something like this is happening?*’ (*Jamie*). Actions were also driven by positive emotion, to provide a sense that ‘*I'm actually doing something here*’, which can ‘*make me feel happy*’ (*Jacqui*). Importantly, participants did not describe these lifestyle changes as inherently distressing or regretted; rather, distress arose when such actions became experienced as morally compulsory, unbounded, or insufficient within a broader context of perceived systemic inaction.

Many participants worked in climate‐related fields, leading to constant exposure to climate discourse across professional and personal domains: ‘*it's what I do for work*…*so I spend the majority of my time thinking about it and doing things in relation to climate and sustainability*’ (*Katie*). Immersion in climate information and climate action often resulted in exhaustion and burnout. Attempts to disengage for self‐preservation conflicted with participants' moral codes, leading some to ‘*feel guilty for not engaging and for feeling like I need to take a break*’ (*Katie*).

#### Constant stress

Participants described somatic experiences similar to those of anxiety and exhaustion stemming from constant thinking about climate change‐related matters: ‘*I feel quite paralysed physically*…*because I'm having all these different thoughts*’ (*Katie*). Participants described their cognitive experience as overwhelming and uncontrollable, and their thoughts as intrusive, ‘*pervasive and insidious*’ (*Dave*).

For me it's the constant…anxiety, the thoughts about what is going wrong with…what the companies and other people are doing to the earth to make profit Billy.

Hypervigilance was a recurring theme, with participants describing their view of the world being completely consumed by CD. For many, this involved heightened sensitivity to environmental cues and distressing information. Common triggers included negative media coverage and observable changes in the environment: ‘*All I see that I'm surrounded with, it's a big word, but death. I'm surrounded with things that are not made in balance with the planet we're living [on]*’ (*Billy*).

Rumination and worry also contributed to CD's all‐consuming nature, with participants caught in unending loops of unresolved thought. Kate described ‘*spiralling*’ due to ‘*not being able to control my thoughts*’. Trying to ‘*counteract a negative thought with a more positive one*’ leads to finding ‘*another kind of thought to counteract the positive one, so it just keeps going and going and going and going*…’.

For some, this evolved into catastrophising – escalating hypothetical scenarios that magnified their sense of fear and uncertainty. Lauren wondered: ‘…*if there's no food security and I'm growing my own tomatoes, will anyone come in my own garden to steal [them]?*’ It pained her that she might need to ‘*fight the intruder*’ even though ‘*this person needs food and I'm stopping them from feeding their family*’.

Whilst some participants identified with catastrophic thinking, others would argue that this wasn't catastrophic or pessimistic; it was realistic: ‘*Am I an optimist? No. Am I a pessimist? No. Am I a realist? Yes*.’ (*Jeff*). However, whilst rooted in real concerns, these kinds of thoughts felt cognitively overwhelming and consuming and kept participants disconnected from the present moment. Being considered a catastrophist by others was also experienced as hurtful.

#### Too much for others

Participants' social lives also became consumed by CD, making big changes to their social circles because of their distress. Many participants described that they no longer wanted to subscribe to social norms in conversation, only wanting to talk about climate change because this is the topic that mattered most to them:

People do not want to talk about this all the time. They wanna talk about their kids, they wanna talk about their dogs…all I wanna talk about is this and people get tired. So, what I have been feeling is that I have been pulling out of my social circles Jeff.

This often led to experiences of invalidation and dismissal from others, being seen as ‘crazy’, overbearing and caring too much because of the intensity at which they speak of and engage with climate change. This profoundly shaped participants social experiences, as many decided to distance themselves from those who perceived them in this way. Participants experienced distress when others' views did not align with theirs: ‘*Sometimes I want to scream at their face, you know*, ‘*you need to care about this*’ (*Jacqui*).

These experiences left participants feeling misunderstood, and increased isolation and loneliness, further amplifying the all‐consuming nature of the distress: One participant explained that becoming distressed about climate change had made him ‘*more lonely*’ and made life ‘*more difficult than it should [be] because it affects my social life*’ (*Jeff*).

### Finding relief from distress

Participants held conflicting beliefs about relieving distress, viewing CD as both unchangeable and essential to their moral identity, while simultaneously struggling to tolerate its psychological and emotional intensity. Although they actively sought to alleviate their distress, these internal contradictions appeared to undermine the effectiveness of their coping strategies.

#### Experience of interventions

Taking medication to manage distress was described as helpful in mitigating overwhelming emotions. However, participants thought that taking medication was unhelpful overall, as less distress demonstrated a misalignment from their values and ideal identities as a caring person, and so some decided to refuse ongoing medication.

Therapy was generally regarded as unhelpful for CD specifically. Participants viewed professionals as unequipped to manage CD: ‘*I think it's like a common feeling that our therapies, they're not able to help us in this*’ (*Beth*). Participants often found therapy for CD to be invalidating. They found the advice, such as taking a break or to ‘*stop thinking about this*’ (Beth) to be unconstructive as it countered their moral code. Seeing CD as unchangeable and necessary appeared to be a barrier to implementing therapeutic advice. However, participants who attended therapy for other concerns, such as health anxiety, were able to take the advice of their therapist and translate it to CD: ‘*I know enough about anxiety, my own anxiety, that I can recognise that that's what's happening and I can recognise that I change my behaviour according to those thoughts*’ (*Lauren*).

Multiple participants spoke about positive experiences in group settings, either developed by a person with lived experience or a professional. Group settings were seen as helpful as it normalised distress and meant that they felt less alone. Groups were generally seen as validating and supportive experiences, helping people to ‘*confront our feelings*’ (Katie) and how they manage their distress.

#### Self‐implemented strategies

Participants most commonly spoke about acting in alignment with their values as a way of mitigating distress; however, the barriers of burnout and perfectionism were present in almost all conversations. Many participants therefore spoke about the need to balance moral commitment with attending to their own wellbeing: ‘*take a break. Start to think in a solution or in an action, in a concrete action. That's helped me*.’ (*Beth*).

Notably, many participants discussed the value of immersive activities promoting awareness of the present moment for shifting focus away from cognitive rumination. For example, one participant described paragliding as a hobby that allowed him not to think about climate change as it demanded his full attention. Participants also sometimes used breathing and grounding exercises, but could find it difficult to engage with these due to the all‐consuming nature of CD.

Participants who expressed an attempt to hold more realistic standards for their own behaviour described less shame and an increased sense of pride and usefulness: ‘*I'd like to think* ‘*you know what? I'm doing what I can*’ (*Ryan*). Hope and optimism were described as essential in managing CD: ‘*I refuse to lose hope*’ (*Lauren*). Participants felt most hopeful when consuming positive climate information or in having productive or compassionate conversations with others.

#### Connection, belonging, and community

The importance of connecting with people who were understanding and empathetic was also referred to throughout most conversations. Participants particularly valued connecting with people who held similar values and beliefs. Interacting with similar people both provided a sense of belonging and validation. Participants particularly valued their CD being validated as rational, rather than ‘crazy’: ‘…*my friends from this [activist] group I think they are able to understand me*… *they don't think that I am intense*…’ (*Beth*). Witnessing people who feel similarly provided participants a sense of relief that they weren't alone in their experiences and that others also held the same morals and acted in alignment with them, providing hope that change was possible.

One complication of connecting with similar people was social comparison. Related to participants' tendency to be self‐critical to achieve moral perfection, comparison of how sustainable they were increased feelings of not being good enough and subsequently distress: ‘*I try not to*… *scroll through my feed*… *[because] I sort of get this information about people's successes*… *in relation to the climate and then feeling like I'm not matching up to that*’ (*Katie*).

## DISCUSSION

This study explored the lived experience of CD, providing new insights into its development, maintenance, and potential alleviation. CD emerged as a morally grounded response to an existential and ongoing global reality, closely tied to participants' values, care for others, and awareness of injustice. While participants experienced this distress as both necessary and overwhelming, they also described its significant psychological costs. These findings support the view that CD is not in itself pathological, but a painful consequence of moral engagement in the context of climate breakdown (Marks & Hickman, 2023).

Central to these findings was the role of moral identity. Participants described CD as life‐altering, enduring, and unchangeable – an integral part of who they were. Participants' experiences of distress shared similarities to those found in moral injury (Litz et al., 2009), whereby individuals became distressed due to the perpetration, failure to prevent, or witnessing of others' actions that transgress sincerely held morals and values. When misalignment with these values occurred, participants often experienced high levels of distress, including shame, anger, and grief. A sense of powerlessness appeared to play a role in CD, whereby the perceived lack of agency intensified feelings of helplessness and led to profound internal conflict about the need for change and the inability to do so. This powerlessness was frequently described in relation to broader systems and institutions perceived as failing to act adequately on climate change. This suggests that participants' distress was not only internally generated but also shaped by a wider socio‐political context in which responsibility was experienced as unevenly distributed, with individuals feeling compelled to compensate for perceived governmental and institutional inaction.

The analysis presents CD as a kind of double‐edged sword which both motivates action and generates relentless internal conflict, with participants viewing distress as morally necessary and fearing that relief would signal disengagement from their values. Relief from CD was therefore often interpreted as acceptance of climate change or letting go of one's beliefs and core values. This presents a clinical dilemma: it is important to acknowledge that distress may be intentionally sustained as a way of remaining morally engaged. Psychological support is therefore complicated when individuals feel ambivalent– simultaneously seeking relief from distress while fearing that its reduction would signal moral disengagement.

It may be important for clinicians to help individuals question the belief that particularly high levels of CD (as seen in this study) are necessary for increased climate action. Ballew et al. (2024) found that experiencing at least a low level of distress can be linked to increased climate action; however, the relationship was found to be non‐linear – higher levels of distress did not lead to more action than low or moderate levels. The results suggest that messages such as these may be better received from therapists who are themselves cognisant of the realities of climate change and are not speaking from a position of minimising concerns.

Responsibility emerged as a central theme in participants' accounts, tightly bound to their moral identity. This aligns with the Value–Belief–Norm model (Stern et al., 1999), which emphasises personal moral obligation as a driver of pro‐environmental behaviour. Responsibility was generally viewed as positive and motivating; however, our findings also highlight ways in which it could become excessive and unsustainable. This internalisation of responsibility must be understood within a broader cultural and political context, rather than solely as an individual cognitive tendency. Public discourse around climate change has frequently framed responsibility at the level of individual behaviour, a process widely critiqued in the environmental psychology and policy literature (Maniates, 2001; Norgaard, 2011). Such framings may inadvertently encourage people to monitor, moralise, and self‐scrutinise their behaviour even when broader structural causes are clearly recognised. Participants noted that inaction from powerful actors such as government and leaders contributed to CD; this political context may intensify the responsibility felt by individuals to take personal action.

At the level of individual psychological processes, internalised responsibility often took a rigid form. Perfectionistic standards, all‐or‐nothing thinking, and beliefs that distress itself was morally necessary (similar to Type II worry beliefs; Wells, 1995) meant that responsibility was often experienced as relentless. In this sense, responsibility overlapped with mechanisms described in clinical models of obsessive‐compulsive disorder (OCD), where inflated responsibility beliefs sustain distress and drive maladaptive behaviours (Salkovskis, 1985). Importantly, the parallel is not exact: unlike OCD (typically characterised by ego‐dystonic intrusions), participants' thoughts about climate change were ego‐syntonic, experienced as consistent with values and even necessary to preserve moral engagement. Nevertheless, techniques used to address inflated responsibility in OCD– such as re‐evaluating responsibility beliefs– may be relevant, provided they are applied sensitively to the climate context. For example, interventions could aim to help individuals explore the possibility that reducing distress or responsibility does not equate to being uncaring or morally disengaged, but may instead allow for more sustainable engagement in a worthy cause.

It is important to note that lifestyle changes such as altering diet, travel, or career choices were not inherently maladaptive. Many participants described these actions as values‐driven, identity‐affirming, and emotionally rewarding. Difficulties arose when such actions became tied to rigid self‐evaluative standards, such that any perceived limitation or inconsistency was interpreted as moral failure rather than a realistic constraint within unjust systems.

Supporting value alignment should be approached with caution in view of the perfectionistic tendencies and binary thinking exemplified by those in the sample. Acting in complete ethical alignment was interpreted as a way of evading moral‐related distress, yet the standards participants set were often demanding and unachievable within existing social structures. While such actions could temporarily relieve distress, they also intensified self‐criticism and shame when perfection was not attained. These findings support Clayton's (2020) argument that CD is often intensified by high moral standards and rigid expectations of actions (overlapping with perfectionistic traits). Pereira et al. (2024) also demonstrated in their empirical study that higher levels of perfectionism were associated with poorer psychological adaptation to climate change. They suggest that interventions aiming to reduce rigid and binary ways of relating to moral standards and responsibility may be beneficial. This resonates with a broader strand of third‐wave and contextual therapeutic approaches, including those that focus on developing mindfulness awareness and acceptance of one's experience and self‐compassion (Hayes et al., 2011). Therapeutic work within these frameworks may help individuals redefine what value‐consistent action looks like under real‐world constraints, exploring the limits of individual responsibility within systemic problems, and supporting engagement that preserves both moral integrity and psychological wellbeing.

From this perspective, distress is not necessarily something to be eliminated, nor are morally driven concerns assumed to be maladaptive. A moral injury framework may provide a useful bridge between therapeutic support and collective action, highlighting how distress can arise from witnessing harm, betrayal by authorities, and the inability to prevent foreseeable suffering, rather than individual vulnerability alone. By locating distress in violations of collective moral obligations and failures of authority, it highlights the role of collective action as a psychologically reparative response. This framing may also help individuals who strive for perfect moral alignment to recognise when responsibility has been unfairly internalised, reducing the unrelenting sense of personal burden without undermining core values or commitment.

Given the moral and responsibility‐based nature of CD, it is unsurprising that its consequences were not only intrapsychic but relational. This study found that CD can significantly strain social relationships and contribute to feelings of loneliness and isolation. This supports findings from recent empirical research (Hajek & König, 2022), which found that higher levels of CD were significantly associated with increased loneliness and perceived isolation due to feeling disconnected from others who do not share their level of concern.

Finding a community of like‐minded people was described by some as an important strategy for coping well with CD as it can improve connection and reduce loneliness, and participants also found validation in spending time with others who felt similarly. In a recent scoping review, Xue et al. (2024) suggest that community‐based interventions, such as climate cafes, could be beneficial in gathering a community of like‐minded people who are experiencing CD and are in need of more social support/connection. Ballew et al. (2024) and Ojala (2012a, 2012b) also discuss the benefits to individuals of engaging in collective climate action, such as participating in protests and community clean‐up projects, as they not only offer relief through moral alignment in taking action but also through fostering a sense of community. The present study's results also suggest that group‐based therapies may be particularly beneficial insofar as there may be more opportunities for validation, social connection, and a sense of collective responsibility.

It important to caveat this with the finding in the present study that, even within supportive groups, comparisons driven by moral perfectionism sometimes intensified self‐criticism and shame, thereby reinforcing distress. This suggests the clinical relevance of increasing individuals' capacity for self‐compassion. It also suggests that climate groups and movements may benefit from attending to how norms, comparisons, and implicit expectations are communicated, to avoid inadvertently exacerbating distress from those who are already highly engaged.

### Strengths, limitations and future research

This study contributes to the emerging literature on CD as, to our knowledge, the first to qualitatively explore its lived experience. It offers novel insights from a clinical psychology perspective, informing conceptualisation and potential case formulation. Crucially, participants' distress cannot be understood solely at the individual level, but must be situated within broader social and political contexts that place disproportionate responsibility on individuals in the face of systemic inaction.

Methodologically, the study has several strengths. Semi‐structured interviews enabled rich, participant‐led accounts, and the homogeneity of the sample in distress levels (as shown by the CC‐DIS) allowed for focused exploration of shared experiences. Reflexivity was maintained through researcher diaries, supervision, and positionality statements.

There were some limitations to the study. Credibility could have been enhanced through member checking and multiple analysts. The interview schedule, grounded in a cognitive‐behavioural framework, may have introduced a Western, clinical lens that narrowed scope. Restricting participation to English speakers likely excluded culturally diverse expressions of CD.

The study intended to recruit individuals who experience CD to the extent that it impacts on their daily lives. Although some level of distress about climate change is not uncommon (Whitmarsh et al., 2022), mean CC‐DIS scores of our participants revealed a sample high up on the CD continuum, and our findings are therefore to those with more extreme levels of this experience. This specificity strengthened the ability to explore experiences in depth but limits generalisability. Nonetheless, as recruitment focused on climate‐engaged/activist channels, our findings are less representative of individuals who are more disengaged.

The sample was limited to working‐age adults. It would be useful to determine whether these findings are generalisable to other groups, including children and young people, older adults, and underrepresented populations, such as those from the Global South and Indigenous communities. A further limitation is that demographic data relating to class and socioeconomic status were not gathered, which would allow for further comment about the applicability of our findings to different groups.

Intervention studies could test the impact of exploring ways of holding responsibility in a way that is self‐compassionate and appreciates the limits of individual agency given their particular socio‐cultural context, and compare group versus individual approaches from both participant and therapist perspectives. Greater attention to moral identity, moral injury, and perfectionism is also needed, both in interventions and in the refinement of measures of CD.

## CONCLUSIONS

This study provides new insights into the lived experience of CD, highlighting its deep entanglement with moral identity, perfectionism, and a persistent sense of inner conflict. CD was found to be both motivating and debilitating, viewed by participants as a necessary expression of moral values, yet also psychologically exhausting and isolating. The findings underscore the importance of not pathologising CD or aiming to eliminate it, but instead, understanding it as a complex, morally‐driven response requiring compassionate, context‐sensitive support. Clinically, this means helping individuals navigate distress without reinforcing perfectionistic or binary thinking, and promoting sustainable value alignment by increasing self‐compassion. Group‐based and community‐oriented interventions may offer particular benefit by providing validation and reducing isolation, though they must be approached carefully to avoid reinforcing comparison and shame. Future research should explore CD across the life‐span and diverse populations, assess the effectiveness of tailored interventions, and further examine the role of moral injury and perfectionism in sustaining distress. By deepening our understanding of CD's moral and relational dimensions, we can better support individuals in living meaningfully and sustainably alongside it.

## AUTHOR CONTRIBUTIONS

**Jessica L. Morgan:** Investigation; data curation; methodology; formal analysis; project administration; writing – original draft; writing – review and editing. **James D. Gregory:** Supervision; writing – review and editing; conceptualization. **Marc O. Williams:** Conceptualization; methodology; validation; supervision; writing – review and editing.

## CONFLICT OF INTEREST STATEMENT

Marc Williams was one of the Editors‐in‐Chief of Psychology and Psychotherapy: Theory, Research and Practice at the time of submission. To avoid any potential conflicts of interest, he had no involvement in the peer‐review process or editorial decision‐making for this manuscript.

## Supporting information


Table S1 and S2:

## Data Availability

Research data are provided as excerpts in the manuscript. Transcripts from the participant interviews and focus groups are not shared.
