# Supplementary material for: ‘Taking the green pill’: An interpretative phenomenological analysis of the lived experiences of climate distress
Source: Psychol Psychother. 2026 Mar 11;99(2):705–20. doi: 10.1111/papt.70052 (PMC13162188; doi:10.1111/papt.70052)
Supplement: Supplementary file 1 — Table S1 and S2: [file PAPT-99-705-s001.docx]

**Table S1.**

*Participant scores of the Climate Distress and Impairment scale (CC-DIS; Hepp et al., 2023; N=10). Items indicated with* ***(R)*** *are reversed scored.*

|  | **CCD Mean** | **CCD SD** | **CCI Mean** | **CCI SD** |
| --- | --- | --- | --- | --- |
| ***Participants*** |  |  |  |  |
| Jamie | 4.46 | 0.76 | 3.625 | 1.06 |
| Beth | 4.87 | 0.26 | 2.25 | 1.28 |
| Dave | 4.4 | 0.83 | 3.5 | 1.2 |
| Ryan | 4.33 | 0.45 | 3.38 | 0.52 |
| Katie | 5 | 0 | 4.5 | 0.76 |
| Billy | 4.73 | 0.46 | 3.13 | 0.83 |
| Jacqui | 4.53 | 0.76 | 3 | 0.53 |
| Jeff | 4.73 | 0.46 | 3.75 | 0.71 |
| Rhi | 4.73 | 0.59 | 2.71 | 0.49 |
| Lauren | 4.73 | 0.46 | 3.13 | 1.13 |
| ***Overall*** | **4.65** | **0.24** | **3.40** | **0.28** |

**Table S2.**

*Summary of CC-DIS scores per item.*

|  | **% of sample** | | | | |
| --- | --- | --- | --- | --- | --- |
| ***Distress Items*** | **Strongly Disagree** | **Disagree** | **Neutral** | **Agree** | **Strongly Agree** |
| *I feel angry when I see how little is done to combat climate change* |  |  |  | 30% | 70% |
| *When I think about climate change, I worry about the future* |  |  |  | 10% | 90% |
| *I am not sad about climate change* ***(R)*** | 80% | 20% |  |  |  |
| *I am enraged that we have missed many chances to stop climate change* |  |  |  | 40% | 60% |
| *I do not fear my future on this planet* ***(R)*** | 60% | 10% | 10% | 20% |  |
| *News about climate change makes me feel depressed* |  |  |  | 50% | 50% |
| *I am not mad when others damage the climate* ***(R)*** | 90% | 10% |  |  |  |
| *The uncertainty about how climate change will progress scares me* |  |  | 10% | 30% | 60% |
| *I feel sad that climate change is causing people and animals to suffer* |  |  |  | 10% | 90% |
| *I do not get upset when others ignore climate change* ***(R)*** | 40% | 50% |  | 10% |  |
| *I am scared that people will lose their homes because of climate change* |  |  |  | 40% | 60% |
| *I feel sad that some parts of the environment will not recover from the effects of climate change.* |  |  |  | 20% | 80% |
| *I am not angry that some countries have missed their climate protection goals* ***(R)*** | 60% | 40% |  |  |  |
| *The impact that climate change has on the planet saddens me* |  |  |  | 40% | 60% |
| *I feel carefree when I think about climate change* ***(R)*** | 80% | 20% |  |  |  |
| ***Impairment Items*** |  |  |  |  |  |
| *Climate change drains all my energy* |  | 20% | 50% | 20% | 10% |
| *My thoughts and feelings about climate change do not affect how well I sleep* ***(R)*** |  | 30% | 40% | 30% |  |
| *When I think about climate change, I get a headache or stomach-ache* | 10% | 60% | 20% |  | 10% |
| *Because of climate change, I am overwhelmed by everyday activities* | 10% | 30% | 20% | 20% | 20% |
| *My thoughts and feelings about climate change do not negatively impact my everyday life* ***(R)*** | 10% | 60% | 30% |  |  |
| *I have no trouble mentally tuning out climate change* ***(R)*** | 50% | 40% | 10% |  |  |
| *Constant discussions about climate change are affecting my relationships* | 10% | 30% | 20% | 20% | 20% |
| *When I think about climate change, I cannot bring myself to work/study* | 10% | 20% | 30% | 40% |  |
